# Radiomics-Based Machine Learning Model for Predicting Overall and Progression-Free Survival in Rare Cancer: A Case Study for Primary CNS Lymphoma Patients

**DOI:** 10.3390/bioengineering10030285

**Published:** 2023-02-22

**Authors:** Michela Destito, Aldo Marzullo, Riccardo Leone, Paolo Zaffino, Sara Steffanoni, Federico Erbella, Francesco Calimeri, Nicoletta Anzalone, Elena De Momi, Andrés J. M. Ferreri, Teresa Calimeri, Maria Francesca Spadea

**Affiliations:** 1 Department of Experimental and Clinical Medicine, University of Catanzaro, 88100 Catanzaro, Italy; 2 Department of Mathematics and Computer Science, University of Calabria, 87036 Rende, Italy; 3 Neuroradiology Unit, IRCCS San Raffaele Scientific Institute, 20132 Milan, Italy; 4 Lymphoma Unit, IRCCS San Raffaele Scientific Institute, 20132 Milan, Italy; 5 Neuroradiology Unit and CERMAC, San Raffaele Scientific Institute, Vita-Salute San Raffaele University, 20132 Milan, Italy; 6 Department of Electronics, Information and Bioengineering, Politecnico of Milan, 20133 Milan, Italy; 7 Institute of Biomedical Engineering, Karlsruhe Institute of Technology (KIT), 76131 Karlsruhe, Germany

**Keywords:** rare tumor, PCNSL, radiomics, image normalization, MRI

## Abstract

Primary Central Nervous System Lymphoma (PCNSL) is an aggressive neoplasm with a poor prognosis. Although therapeutic progresses have significantly improved Overall Survival (OS), a number of patients do not respond to HD–MTX-based chemotherapy (15–25%) or experience relapse (25–50%) after an initial response. The reasons underlying this poor response to therapy are unknown. Thus, there is an urgent need to develop improved predictive models for PCNSL. In this study, we investigated whether radiomics features can improve outcome prediction in patients with PCNSL. A total of 80 patients diagnosed with PCNSL were enrolled. A patient sub-group, with complete Magnetic Resonance Imaging (MRI) series, were selected for the stratification analysis. Following radiomics feature extraction and selection, different Machine Learning (ML) models were tested for OS and Progression-free Survival (PFS) prediction. To assess the stability of the selected features, images from 23 patients scanned at three different time points were used to compute the Interclass Correlation Coefficient (ICC) and to evaluate the reproducibility of each feature for both original and normalized images. Features extracted from Z-score normalized images were significantly more stable than those extracted from non-normalized images with an improvement of about 38% on average (*p*-value < $10^{-12}$). The area under the ROC curve (AUC) showed that radiomics-based prediction overcame prediction based on current clinical prognostic factors with an improvement of 23% for OS and 50% for PFS, respectively. These results indicate that radiomics features extracted from normalized MR images can improve prognosis stratification of PCNSL patients and pave the way for further study on its potential role to drive treatment choice.

## 1. Introduction

Primary diffuse large B-cell lymphoma (DLBCL) of the central nervous system (CNS) (PCNSL) is a rare form of aggressive extranodal non-Hodgkin’s lymphoma limited to the CNS and, thus, potentially involving the brain, spinal cord, meninges, and eyes [1,2]. Magnetic resonance imaging (MRI) before and after contrast injection is the recommended imaging modality in the case of PCNSL suspicion and for disease staging after diagnosis confirmation by histopathological examination of a tumor biopsy [3]. The modern treatment of PCNSL is based on two phases, induction and consolidation [3,4]. The first one typically consists of high-dose methotrexate (MTX)-based chemotherapy, while the second one may include several options, among which high-dose chemotherapy, followed by autologous stem cell transplantation (HCT–ASCT), is presently the golden standard [5,6,7]. Although new therapeutic approaches have improved overall survival [5,8], about 30% of patients <70 years are primary refractory to HD–MTX-based chemotherapy and nearly 25% of patients relapse after consolidation [9]. Unfortunately, the reasons underlying this poor response to therapy are not known. Nevertheless, being able to identify, in advance, patients who are going to respond to the current treatment would be of the uttermost importance, as it may help in driving clinical decision making and in tailoring treatment accordingly.

Radiomics is a computational technique to extract high-dimensional quantitative features from medical images [10], which embed information about shape, intensity, and texture of a particular Volume of Interest (VoI). It assumes that medical images reflect underlying characteristics of disease-specific pathological processes and quantitative analysis can objectively capture and describe such mechanisms [11]. In recent years, the application of Artificial Intelligence (AI) techniques in the biomedical field [12,13] has been rapidly expanding. Advanced analytical and machine learning (ML) tools with radiomics features [14] have been used to improve diagnosis [15], or to allow prognostic stratification [16] and customization of therapy in oncology [17]. In contrast to a traditional biopsy, which is limited to the analysis of a small amount of tissue sample, one of the advantages of Radiomics is the possibility to characterize the whole tumor volume, and, thus, capturing extended lesion properties, such as size, shape and heterogeneity, or changes over time on image series [18]. Several radiomics studies have so far been conducted for highly prevalent common cancer types, such as lung [19], breast [20], and colon [21]. However, for rarer cancer types, especially for PCNSL, the literature is still very limited. In this context, studies have mainly focused on differentiating PCNSL from glioblastoma (GBM) [22,23,24,25,26,27] starting from multi-parametric MRI [22,28]. On the other hand, the correlation between radiomics features and therapy response or outcome has been barely investigated for PCNSL [29]. Chen et al. [30] evaluated the prognostic value of radiomics features for predicting Overall Survival (OS) in 52 PCNSL patients. However, the study was limited only to the analysis of textural features on contrast enhanced MRI. Ale et al. [31] carried out a predictive analysis on OS and Progression-Free Survival (PFS) considering a population of 47 patients, respectively. Promising results were achieved, although few details about the methodology and the patient cohort were provided. A schematic overview about the State of Art (SoA) of PCNSL and Radiomics Analysis is given in Table S1 in the Supplementary Data. A common problem for studies related to PCNSL is that recruiting patients with such a disease in a single center may be difficult, due to the relatively low incidence of the tumor [32]. Nonetheless, some issues must be taken into account for radiomics data deriving from multiple institutions. Inter- and intra-scanner variability is a common problem for multicenter MRI studies and, for this reason, the normalization of the intensity of the gray level becomes of fundamental importance in radiomics analyses.

Herein, we report a machine learning-based approach for predicting one-year OS and PFS in patients with PCNSL undergoing treatment with a high-dose methotrexate-based chemotherapy regimen. The proposed method relies on extracting robust and stable radiomics features from MRI scans. Such robustness and stability was assessed by comparing different intensity normalization methods on patient images acquired at different time points. To our knowledge, only a few studies have investigated the importance of image normalization in radiomics studies, despite it constituting an important challenge when using MRI data. In fact, the definition of a protocol is still missing [33,34,35,36,37]. Moreover, to date, the role of image normalization for radiomics analysis of PCNSL tumors has not yet been evaluated.

## 2. Materials and Methods

### 2.1. Dataset Description

**Figure 1:**
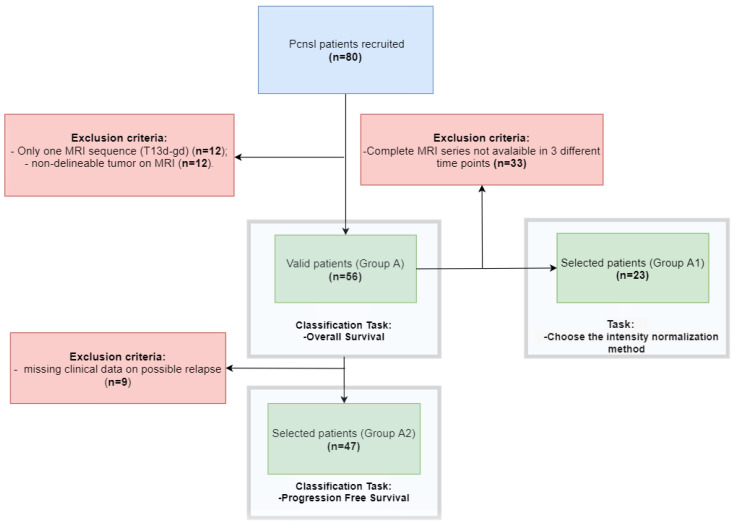
Flowchart of the patient enrolment process. In the blue box, the initial number of patients available for this study. In the red box, the reasons for exclusion of some patients (unavailability of complete MRI sequences or missing clinical data). In the green box, the number of patients selected for the specific task.

Clinical and MRI data from 80 patients with histological or cytological diagnosis of PCNSL, as well as absence of extra-CNS disease as per international guidelines [38], treated at San Raffaele Scientific Institute of Milano, Italy, between January, 2010, and November, 2019, were retrospectively collected. MRIs were acquired in different centers and with different scanners. Patients were considered eligible for subsequent analyses based on the following criteria (see Figure 1): (1) availability of T1-W, T2-W, Fluid Attenuated Inversion Recovery (FLAIR) and T1-W with gadolinium (T1 gd) pulse sequences on MR scans obtained before the start of therapy; (2) tumor contours clearly distinguishable for manual segmentation. Overall, 56 patients were included for the OS classification (Group A) and 47 patients (Group A2) for PFS. From Group A, 23 patients (Group A1) were imaged at 3 different time points (before, during and after the treatment) and with different scanners (described for each group in Table S2 in Supplementary Data) were selected for feature stability analysis. The demographics and clinical features of the patient cohort are summarized in Table 1. This observational study was approved by the Ethical Committee of San Raffaele Hospital in Milan (Italy) with number 22/INT/2021 and conducted in accordance with all international laws and rules, and in accordance with the national laws, as well as in accordance with all applicable guidelines. Due to the retrospective nature of this study and anonymized clinical data, ad hoc informed consent was waived.

### 2.2. Image Pre-Processing

**Figure 2:**
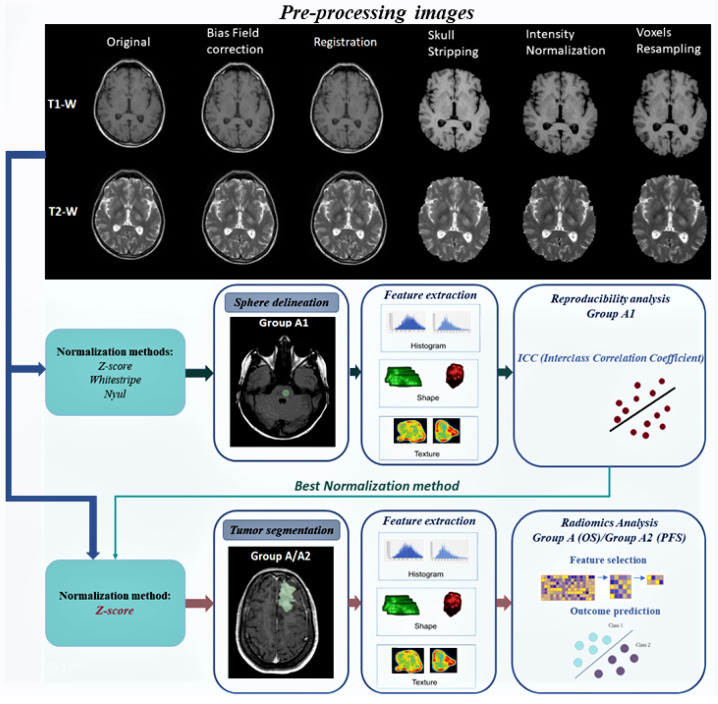
The workflow of the study was divided into two main sections: (1) Reproducibility analysis of features extracted from pathological tissue of MR images normalized with three different methods (Z-score, WhiteStripe and Nyul); (2) Radiomics Analysis for predictive OS and PFS of PCNSL patients (features extracted from segmentation tumor). For both sections, the first step was to pre-process MRI sequences. From the results of the reproducibility of features, the Z-score method was selected for application to the MRI sequences.

All images were pre-processed according to the steps described below (see Figure 2), in order to improve their quality and to increase the reproducibility of radiomics features [39]:

1. to correct the non-homogeneous intensity of the magnetic field present in MR images, the module “N4ITK MRI bias correction” available in 3D Slicer [40] was used [41];
2. for each patient, all available MRI acquisitions were registered on the T1-gd image (sequence where segmentation was performed);
3. skull stripping [42] was performed from images to remove extra brain tissue from the brain volume and to increase the accuracy of subsequent MRI processing. The “Swiss skull stripper” module of 3D Slicer was used [43];
4. normalization methods were applied for MRI intensities normalization (described in detail in Section 2.2.1);
5. all sequences were resampled (voxels 1 mm${}^{3}$) [44].

#### 2.2.1. Intensity Normalization of MR Images

Three gray level intensity normalization methods were tested on the MR images: Z-score, WhiteStripe and Nyul.

The Z-score method normalizes the image I(x) by subtracting the mean of the image $\mu_{brain}$ and dividing by the standard deviation of all the voxel intensities $\sigma_{brain}$:

(1) $$I_{Zscore}\left(x\right)=\frac{\left(I\left(x\right)-\mu_{brain}\right)}{\sigma_{brain}}$$

The WhiteStripe method [45] was developed to bring raw image intensities to a biologically interpretable intensity scale. The method applies a z-score transformation to the whole brain using parameters estimated from a latent subdistribution of normal-appearing white matter (NAWM). In detail, this method normalizes the image I(x) intensities by subtracting $\mu_{ws}$, which corresponds to the mean intensity value of the (NAWM), from each voxel intensity I(x) and dividing the result by the standard deviation of the NAWM $\sigma_{ws}$:

(2) $$I_{ws}\left(x\right)=\frac{\left(I\left(x\right)-\mu_{ws}\right)}{\sigma_{ws}}$$

The method developed from Nyul et al. [46], also called piecewise linear histogram matching normalization, learns a standard image histogram from a set of images, and then linearly maps the intensities of each image to this standard image histogram. MRI intensities are not standardized. For this reason, before carrying out Radiomics analyses, the intensity normalization of the gray levels of images is essential.

The code used for this implementation is available at https://github.com/jcreinhold/intensity-normalization (accessed on 19 February 2023).

### 2.3. Segmentation VOI (Volume of Interest) and Features Extraction

The hyperintense tumor lesion on post-contrast T1-W images was manually segmented for each patient resulting in volume of interest (VOI). The same VOI was reported in the other sequences for each patient applying the linear transformation identified by the registration process. All segmentations were performed by R.L., a radiologist with 4 years of experience, at the time of the study. Radiomics features were extracted from the VOI using Pyradiomics 3.0.1 (https://pyradiomics.readthedocs.io/en/latest/features.html, (accessed on 19 February 2023) [47]: 19 First Order (F0) features, 14 Shape features, 23 Gray Level Co-I Matrix (GLCM) features, 16 Gray Level Run Length Matrix (GLRLM) features, 16 Gray Level Size Zone Matrix (GLSZM) features and 14 Gray Level Dependence Matrix (GLDM) features [48]. In total, 120 features (including radiological features) were extracted from the tumor region of each MRI sequence from both non-normalized images and normalized images with the chosen method.

### 2.4. Machine Learning Model Building

Given as input a set of radiomics features extracted from processed MRIs (Group A), the goal was to train a machine learning model to predict the probability of survival of a patient with PCNSL. Since the prediction task had only two possible outcomes (survive/not survive after 1 year), the task was modeled as a binary classification problem. A first selection of the features was performed, using a high correlation filter to remove variables having large absolute correlation. To overcome the curse of dimensionality issues and reduce overfitting, the Min–Max Normalization method was applied to linearly transform radiomics features by using scikit-learn library (https://scikit-learn.org/stable/modules/generated/sklearn.preprocessing.MinMaxScaler.html, accessed on 19 February 2023). Only relevant features were selected in cross validation according to an ensemble of four selection methods: (i) SelectKBest for the chi-square test method; (ii) the Recursive Feature Elimination (RFE) using the Logistic Regression model; (iii) least absolute shrinkage and selection operator (Lasso), and (iv) Select From Model using RandomForestClassifier model. In detail, each method extracted k=15 candidate features and only the ones selected by at least three algorithms over four were chosen to feed the classification algorithm. Five classifiers were tested, namely: Extra Tree Classifier (ETC), Support Vector Machine (SVM), Logistic Regression (LR), Random Forest (RF), kNeighbors (KN). Feature selection methods and ML classifiers were implemented, based on the scikit-learn library version 0.23. The whole process, from the normalization of features to the selection and classification, was performed in a repeated five-fold stratified cross-validation (https://scikit-learn.org/stable/modules/generated/sklearn.model_selection.RepeatedStratifiedKFold.html, accessed on 19 February 2023) (10 repetitions) was adopted to assess overfitting and to evaluate the stability of the results. The workflow of this study is described in Figure 2.

### 2.5. Experiments

#### 2.5.1. Feature Robustness

To determine which normalization method was best suited for our dataset, we studied the effect of image intensity normalization on the reproducibility of the radiomics features. To this aim, Group A1 (subgroup of Group A, as described in Section 2.1 and shown in Figure 1) was considered. Notice that the selected subgroup of patients was not considered during the survival prediction analysis, in order to avoid any bias in the classification results.

Given a patient, all his/her longitudinal T1-W and T2-W sequences were, in turn, normalized using three methods: Z-score, WhiteStripe and Nyul (described in Section 2.2.1). Then, a region of the pons, where no pathological modifications were observed, was identified on the patient’s FLAIR image. From this region, a 1 cm diameter spherical segmentation was extracted using the segmentation tool of 3D Slicer software. The segmentation was reported for all the longitudinal sequences of the patient by applying the linear transformation of the registration between the images made previously. A total of 94 radiomics features were extracted with the Pyradiomics library. Shape features were excluded as the selected spherical VOI was equal for all patients. For the three longitudinal acquisitions of each patient, we extracted features from images normalized with three methods previously described and from the non-normalized images for sequences T1-W and T2-W.

The Interclass Correlation Coefficient (ICC) was calculated to evaluate the reproducibility of each feature for each normalization method. Formally, the ICC is a descriptive statistic that can be used when quantitative measurements are made on units organized into groups [49]. It ranges between 0 and 1, indicating null and perfect reproducibility. ICCs were calculated with IBM’s SPSS statistical software, using the two-way random mean measurement ICC (2,k). We defined a matrix nxk, with n number of features extracted for each patient and k, number of observers (i.e., MRI acquired with different scanners). Given MSr the average square for rows, MSe the residual average, and MSc the average square for columns:

(3) $$ICC\left(2,k\right)=\frac{\left(MS_{r}-MS_{e}\right)}{MS_{r}+\frac{\left(MS_{c}-MS_{e}\right)}{k}}$$

ICCs were computed to assess the stability of first-order and textural features across the three acquisitions before and after normalization. The Kruskal–Wallis test and its post hoc were used to compare the obtained ICCs for T1-w and T2-w sequences, under the assumption that data were not normally distributed. The best normalization method was applied to images of groups A/A2 for subsequent Radiomics analysis.

#### 2.5.2. Overall and Progression Free Survival Prediction

Patients were dichotomized, based on OS or PFS greater than, or lower than, 12 months, respectively. OS was defined as time from diagnosis until death due to any cause or date of last follow-up visit, and PFS was defined as time from diagnosis until progression, relapse, death or date of last follow-up visit [50].

Each of the selected ML algorithms was trained at classifying OS for patients in Group A. Classification performances were evaluated in terms of F1-score (https://scikit-learn.org/stable/modules/generated/sklearn.metrics.f1_score.html, accessed on 19 February 2023) and Area Under ROC curve (AUC) (https://scikit-learn.org/stable/modules/generated/sklearn.metrics.auc.html, accessed on 19 February 2023). It is worth noticing that machine learning model validation is a crucial step, especially in the biomedical domain. We also compared the performance of the classifiers using both radiomics features alone as well as combined with clinical features. Age > 60, PS > 2, LDH > ULN, protein CSF > ULN and deep lesion were considered as clinical features, these being considered as available and validating PCNSL risk scores [51,52].

To better evaluate the impact of normalization on survival prediction, each algorithm was trained and tested using radiomics features obtained either from raw or normalized images. Only the Z-score method was used in these experiments since, as shown in Section 3.1, it provided the most stable features.

## 3. Results

### 3.1. Impact of the Intensity Normalization Method on Radiomics Feature

**Figure 3:**
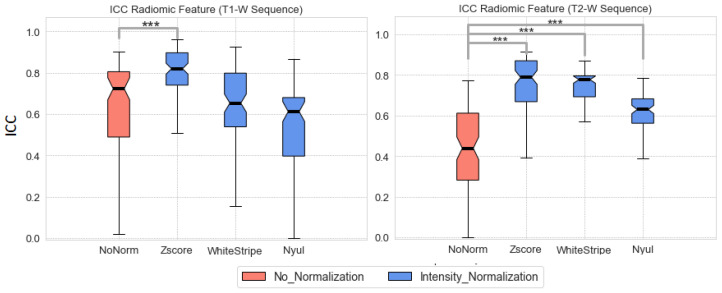
The distribution of ICC values computed from extracted features for non-normalized images and for normalized images with Z-score, WhiteStripe and Nyul methods. *** (significant statical difference).

Figure 3 shows median ± quartiles of ICCs computed on both original and normalized images in T1-W and T2-W Sequences (Group A1). Z-score normalization determined the highest increase in ICC for features extracted from T1-W (30% average increase compared with non-normalized sequences, $p<10^{-9}$). No statistically significant differences were observed when comparing non-normalized T1-W sequences with Nyul or WhiteStripe normalized sequences. All three normalization methods showed a clear increase of ICC values in T2-W sequence (Kruskal–Wallis test, $p<10^{-12}$ (Z-score), $p<10^{-13}$ (WhiteStripe), $p<10^{-15}$ (Nyul).

### 3.2. Performance Comparison of Classification Models

The results of median and quartiles of the F1-scores obtained from the five selected machine learning models for the OS and PFS prediction classification tasks are reported in Table 2. For both tasks, we performed the classification with radiomics features alone, radiomics features + clinical features and clinical features alone.

#### 3.2.1. OS Classification Task

For features extracted from T1-W, T2-W and the combination of T1-W and T2-W features (T1-W/T2-W), classification results obtained from images normalized with Z-score are presented in this section (providing the best results in terms of reproducibility and stability compared to the other normalization methods, as reported in Section 3.1).

Considering only radiomics features, the best performances of T1-W sequence were obtained from classifiers SVM and LR with the median and quartiles, respectively, F1 = 0.77 (0.68–0.83) and F1 = 0.77 (0.73–0.83). For T2-W sequence, the best performances were obtained by the SVM classifier with F1 = 0.80 (0.77–0.86) and LR with F1 = 0.80 (0.75–0.86). For T1-W/T2-W, the performance improved and we obtained a median of F1-score equal to 0.83 (0.77–0.86) with RF classifier. The best results were obtained from normalized images, with a significant statistical difference from the results obtained using features extracted from non-normalized images.

When introducing clinical features, the results did not significantly change. In this case, the best performances were obtained with T1-W (KN = 0.82 (0.73–0.86) and T1-W/T2-W (ETC = 0.80 (0.73–0.86)). Instead, The F1-score for predicting OS using only clinical features was 0.71 (0.66–0.79) with the SVM classifier.

**Figure 4:**
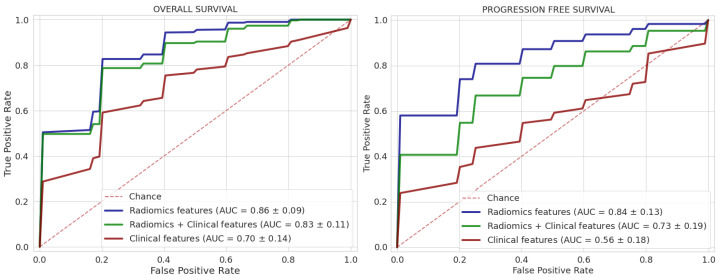
Roc curves of the best classifiers for each feature category: only radiomics features in blue, radiomics + clinical features in green and only clinical features in red (currently validated).

Figure 4 shows the ROC curves of the best performances of classifiers. The AUC values of radiomics features alone, radiomics + clinical features and clinical features alone for predicting OS were 0.86 ± 0.09, 0.83 ± 0.11 and 0.70 ± 0.14, respectively. In comparing the best performance for OS prediction with clinical features, and with radiomics features a significant statistical difference (*p* < $10^{-9}$) was found. There was no significant statistical difference between performance with radiomics features alone, and with Radiomics plus clinical features (*p* = 0.38).

#### 3.2.2. PFS Classification Task

Patients of Group A2 were considered to assess PFS classification task. The patients’ characteristics are summarized in Section 2.1. Considering radiomics features alone, the best performances were obtained from the sequence T2-W (SVM = 0.80 (0.67–0.88) and LR = 0.80 (0.67–0.86)) and from T1-W/T2-W (LR = 0.73 (0.62–0.80)). For the PFS, the combination of T1-W and T2-W sequences did not improve the performance of the model (compared to single sequence).

The addition of clinical features for PFS did not improve performances and, considering only clinical features, the best result was LR = 0.67 (0.63–0.71). Compared to OS prediction, in this case also the best performance was obtained with normalized images for the sequence T2-W and T1-W/T2-W with a statistical difference with non-normalized images.

ROC curves of the best performances for PFS classification (Figure 4) also showed the prediction of radiomics features (AUC = 0.84 ± 0.13) in respect to clinical features (AUC = 0.56 ± 0.18) with a significant statistical difference (*p*-value < $10^{-12}$). There was also a statistical difference between the prediction with radiomics features alone and with the addition of clinical features (*p* = 0.002).

### 3.3. Feature Importance

Beyond the classification scores, further analyses were conducted to better understand the role of the features in the classification process. The study was performed for each imaging modality, with and without intensity normalization. We considered the RF classifier, where the feature importance was computed as the mean and standard deviation of accumulation of the impurity decreased within each tree of the forest. In more detail, for each independent training in the cross-validation procedure, we ranked the features according to importance score and selected the top 15 (top-15). Then, for each feature, we calculated the frequency with which that feature was selected as top-15 and, from the resulting distribution, we selected the top 13 features for analysis. Simply put, we selected the top 13 features most often ranked as “most important" in each independent training of the cross-validation procedure.

**Figure 5:**
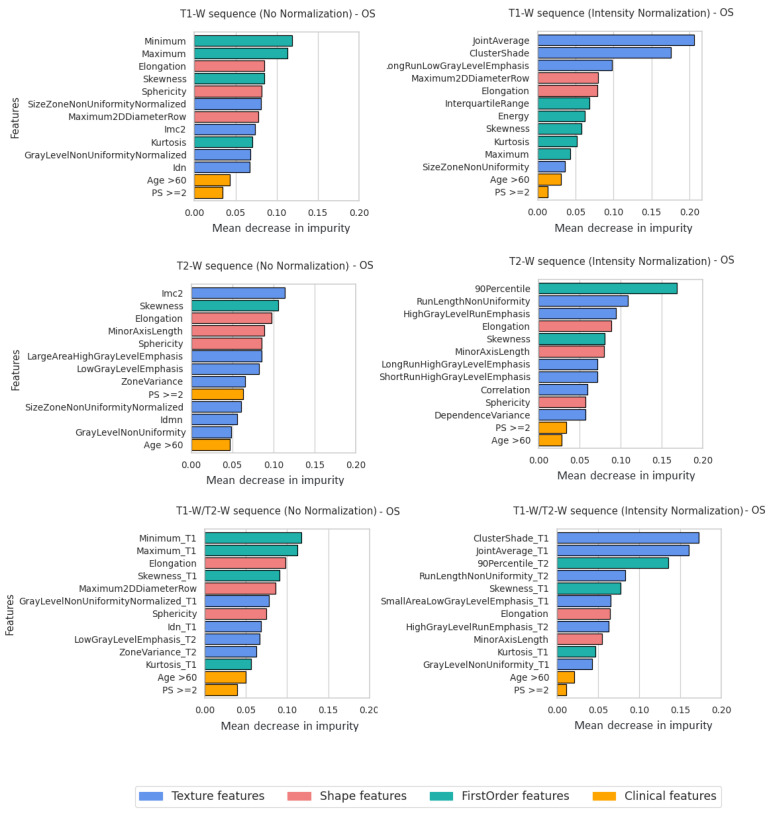
Feature importance for all MRIsequences with and without normalization (OS classification task). Features were grouped using different colors for shape features, texture features, first order features and clinical features.

**Figure 6:**
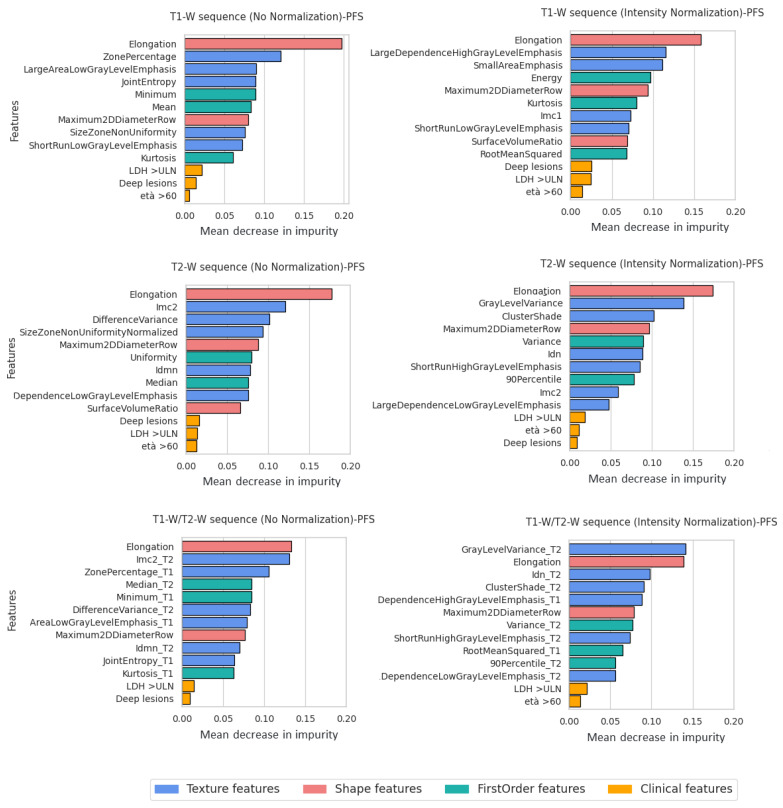
Feature importance for all MRI sequences with and without normalization (PFS classification task). Features were grouped using different colors for shape features, texture features, first order features and clinical features.

Figure 5 and Figure 6 represent the selected clinical and radiomics features for T1-W, T2-W sequences, and T1-W/T2-W sequences. As per the OS classification task, the most selected clinical features were Age and Performance status (PS) (Figure 5), while for the PFS classification task, LDH>ULN, deep lesion, and Age were almost always selected (Figure 6). Considering the feature importance score, radiomics features seemed to give a greater contribution to the outcome than clinical features.

For T1-W and T2-W sequences (without intensity normalization) in the OS classification, the most important contribution was given by shape features (Elongation and Sphericity) and first order features (https://pyradiomics.readthedocs.io/en/1.1.1/features.html#radiomics-firstorder-label, accessed on 19 February 2023) (Minimum, Maximum and Skeweness). For T1-W and T2-W sequences (with intensity normalization), GLCM features (Cluster Shade, Joint Average) and GLRLM features (Long Run Low Gray Level Emphasis, Run Length Non-Uniformity and High Gray Level Run Emphasis ) received the highest scores.

Considering the PFS classification task, an important role seemed to be played by Elongation (shape feature), that shows the relationship between the two largest principal components in the ROI shape, and its value, ranging from 0 (line-like object) to 1 (circle-like object).

(4) $$Elongation=\sqrt{\frac{\lambda_{minor}}{\lambda_{mayor}}}$$

Here, $\lambda_{mayor}$ and $\lambda_{minor}$ were the lengths of the largest and second largest principal component axes. Amongst the selected, we also found Zone Percentage (GLSZM) and Imc2 (GLCM) for non-normalized images, and, concerning normalized images, Large Dependence High Gray Level Emphasis (GLDM) for T1-W and Gray Level Emphasis (GLSZM) for T2-W.

## 4. Discussion

To the best of our knowledge, this is the first study investigating the capability of radiomics features as outcome predictors in patients with newly diagnosed PCNSL, while also evaluating the impact of MR image normalization [53] on feature stability. To overcome the curse of dimensionality issues and to reduce overfitting, feature selection was performed by using multiple approaches and reaching consensus by a voting procedure. A post-hoc analysis of the most salient features learned by the selected ML models was performed, with the aim of trying to collect more insight about the pathology and to partially explain the classification process.

Significant results were obtained for both OS and PFS prediction using all the selected classifiers with a statistically significant difference (*p*-value < $10^{-4}$) between image intensity normalization and no normalization (best median F1-score 0.83 vs. 0.71 for OS and 0.80 vs. 0.71 for PFS, respectively). Interestingly, it was observed that combining features from both T1-W and T2-W sequences improved results in the OS classification task compared to using features from a single sequence. On the other hand, the best performance for PFS was obtained using only the T2-W sequence (median F1-score T1-W/T2-W = 0.73 (0.62–0.80) vs. T1-W = 0.68 (0.66–0.73) vs. T2-W = 0.80 (0.67–0.88)). Noteworthy was the fact that introduction of clinical features commonly used to calculate the IELSG score (age, PS, deep lesions, CSF protein, and LDH) marginally improved the performance of some classifiers only in OS analysis. However, their contribution did not have a significant impact. AUC scores achieved by the best classifiers (RF for OS and SVM for PFS) were observed to be significantly higher compared with scores obtained using only clinical features (*p*-value < $10^{-9}$ and *p*-value < $10^{-12}$, respectively), showing that radiomics features better contributed to the outcome prediction than clinical features. This work has some limitations that are worth mentioning. First, the relatively small number of patients, mainly due to the low incidence rate of PCNSL [32], which could highly impact the learning process and might cause sub-optimal prediction performances and overfitting. Obviously, we resorted to numerous techniques to mitigate the effect of the low number of patients, but, in any case, our future goal is to increase the dataset in order to validate these promising results. Furthermore, images from multiple centers were collected to mitigate the issue and a repeated cross-validation approach was used to evaluate the robustness of our results. Furthermore, up to 30% of the initial study population could not be considered eligible for this study because of lack of MR sequences or delineable tumor. However, we believe this number could be reduced in future radiomics studies in the PCNSL setting, given increasing use of stereotactic biopsy instead of surgery for diagnosis, as well as the potential availability of pre-biopsy MRI scans which could also reduce other technical problems, such as bleeding. Moreover, the recent IPCG (International Primary CNS Lymphoma Collaborative Group) recommendations for MRI imaging should, potentially, also impact on the homogeneity of future studies [54]. Second, our models processed the radiomics features representing the tumor, excluding the possible prediction capability of extra lesion tissues as well as the association between radiomics features and pathological/molecular characteristics, which might reveal hidden relations useful to better understand the history of the disease. Third, information about the performed treatment was not included in the prediction process of the final analysis, as it differed from the main focus of this study. However, up to 93% of patients received an HD–MTX based treatment with a subsequent consolidation/maintenance in nearly 50%, unless there was progression or death due to lymphoma or other causes and, overall, all patients received the best available treatment based on clinical stratification. Further investigation is needed to use this integrated clinical and radiomics approach to stratify patients for therapy response prediction. This would allow not only the division of patients into risk groups, but also definition of the better potential treatment to be studied in future clinical trials.

Furthermore, some aspects of this trial merit discussion. The analyses were performed on the features extracted from T1-W and T2-W sequence and not from the T1 as contrast, as we did not want the radiomics features to be affected by the contrast. All analyses were also carried out on the FLAIR sequence, but the data were not reported in this paper as the results were not satisfactory. We plan to consider it again in future work where deep learning-based models will be explored.

Almost all the work related to this rare tumor has been focused, to date, on the differentiation of PCNSL from atypical glioblastoma [23,24,28]. Instead, in the present study, we evaluated the prognostic value of images normalization to use radiomics features for predicting OS and PFS in PCNSL. Indeed, for rare tumors, one of the limitations is to collect a sufficient quantity of patient data to analyze; thus, assembling data from different centers is usually a valid solution. However, in the case of MRI acquired in a multicenter setting, inter- and intra-scanner variability can be an important limitation in the radiomics analysis. Thus, the study of the effect of normalization on both task prediction and reproducibility of radiomics features is of important value. To this end, a subgroup of patients with three longitudinal acquisitions over time was selected and the ICC for each radiomics features was computed in non-pathological tissue. Three state-of-the-art normalization methods were tested (Z-score, WhiteStripe and Nyul), according to many MR image harmonization studies [33,34,55]. While a similar study performed for Glioblastoma [53] found the Nyul method to be the most robust for radiomics analysis, for MRI of PCNSL patients we found that the Z-score normalization gave the highest number of reproducible features (median and quartile values of all ICCs = 0.8 (0.74–0.90)) for both the T1-W and the T2-W sequences, as shown in Figure 4. Furthermore, in contrast with [53], we performed a feature stability analysis on a portion of healthy tissue so that the results were unaffected by disease progression or regression.

The normalization step had a significant impact on the learning process for both OS and PFS (all results summarized in Table 2). Figure 5 and Figure 6 show the feature importance for each sequence at inference time. As is observable, first order features had the highest importance among the features extracted from non-normalized images. By contrast, when using normalized images, the classifiers seemed to rely more on textural features (GLCM and GLRLM). First order statistics describe the distribution of individual voxel values without concern for spatial relationships. Instead, textural features are obtained calculating the statistical inter-relationships between neighbouring voxels (hence, they provide a measure of intra-lesion heterogeneity) [17]. We speculate that the latter may contain more robust and informative content for the survival prediction, therefore explaining the better classification results. Indeed, textural analysis derived from conventional sequences reflects histopathology features in solid cancer and has been proposed as a novel noninvasive modality to further characterize tumors in clinical oncology [56,57]. Furthermore, it is worth noticing that shape features may also act as confounding factors. If spurious correlation exists (e.g., between tumor size and disease progression) the learning process may be biased. In this case, elongation was the most important feature for almost all sequences and there seemed to be no difference between normalized and non-normalized images, but that was because the shape features were not affected by intensity normalization and depended only on tumor segmentation. Furthermore, the performance improved significantly for the prediction classification task, especially for the T2-W sequence. Probably, the other textural features made the difference. Finally, for the OS survival classification, features of both sequences (T1-W and T2-W) were equally important. The PFS features of the T2-W sequence provided a greater contribution and, in fact, the performance results were better than for the T1-W sequence.

## 5. Conclusions

This work presented the effect of normalization of MR images on a radiomic-based approach to predict OS and PFS in PCNSL patients. Despite the limited number of cases (mainly due to the rarity of the tumor), the proposed method made a breakthrough in radiomics-based precision medicine for PCNSL patients.

## Figures and Tables

**Table 1:** Description of the patient dataset (Group A).

**Eligible Patients (#)**	56/80 (70%)
Male:Female	0.56
Median Age	69 (41–85)
Multiple lesions	32 (58%)
Involvement of deep areas §	45 (80%)
Lactic dehydrogenase serum level >ULN	35 (52%)
Cerebrospinal-fluid protein concentration >ULN *	34(60%)
ECOG—Performance Status >2	30 (53%)
**IELSG risk score**	
-Low	5 (9%)
-Intermediate	28 (50%)
-High	23 (41%)
**Sites of disease**	
-Brain parenchyma	56 (100%)
**Treatment details**	
**Induction**	
MATRix	37 (66%)
MAT	2 (3%)
HD-MTX + HD-ARAC	10 (17%)
HD-MTX + Alkylators	4 (7%)
WBRT ± TMZ	4 (7%)
Rituximab	43 (77%)
**Consolidations**	
ASCT	15 (27%)
WBRT	6 (11%)
DeVIC	5 (9%)
Oral Maintenance	3 (5%)
None	26 (46%)
Unknown	1(2%)
Treatment delay >20 gg	40 (71%)
Refractory to first line ^@^	22 (39%)
1-year PFS	24/47 (51%)
1-year OS	30/56 (54%)

* Lumbar puncture was contraindicated in 3 patients; CSF protein concentration was considered an unfavorable feature in IELSG risk score in these patients. § At least one of the following brain structures: periventricular regions, basal ganglia, corpus callosum, brainstem, and cerebellum. ^@^ PD < 6 months from the end of first line treatment; HD-ARAC: high dose Cytarabine; ASCT: autologous stem cell transplantation; DeVIC: Dexamethasone, Etoposide, Ifosfamide and Carboplatin; ECOG—PS: Eastern Cooperative Oncology Group—Performance Status; HD-MTX: High dose Methotrexate; IELSG: International Extranodal Lymphoma Study Group; LDH: Lactic dehydrogenase serum level; MATRix: High dose Methotrexate, high dose Cytarabine, Thiotepa and Rituximab; pCSF: Cerebrospinal-fluid protein concentration; PFS: Progression free survival; OS: Overall Survival; TMZ: Temozolomide; ULN: upper limit normal; WBRT: Whole brain radiation therapy.

**Table 2:** Median ± quartiles of the F1-Score (T1-W and T2-W and combination T1-W and T2-W), obtained using all 5 test folds with 10 repeated of the cross-validation and 5 machine learning models. The difference between the quartiles provided information on the distribution of results. Each result was compared with features extracted from non-normalized images and normalized images using only radiomics features, radiomics plus clinical features and only clinical features.

**OS**	**Radiomics Features**	**ETC**	**SVM**	**LR**	**RF**	**KN**
**T1-W**	No Normalizazion	0.67 (0.61–0.79)	0.71 (0.70–0.71)	0.71 (0.67–0.71)	0.67 (0.61–0.72)	0.67 (0.61–0.73)
	Intensity Normalization	0.75 (0.67–0.83)	0.77 (0.68–0.83)	0.77 (0.73–0.83)	0.73 (0.67–0.83)	0.73 (0.63–0.80)
**T2-W**	No Normalization	0.67 (0.55–0.73)	0.67 (0.57–0.71)	0.71 (0.67–0.71)	0.59 (0.50–0.71)	0.57 (0.44–0.70)
	Intensity Normalization	0.79 (0.73–0.86)	0.80 (0.77–0.86)	0.80 (0.75–0.86)	0.73 (0.67–0.830)	0.77 (0.72–0.80)
**T1-W/T2-W**	No Normalization	0.67 (0.57–0.72)	0.67 (0.55–0.76)	0.67 (0.60–0.76)	0.61 (0.54–0.71)	0.61 (0.54–0.70)
	Intensity Normalization	0.80 (0.77–0.86)	0.80 (0.72–0.83)	0.80 (0.73–0.83)	**0.83 (0.77–0.86)**	0.80 (0.72–0.83)
**OS**	**Radiomics + Clinical Features**	**ETC**	**SVM**	**LR**	**RF**	**KN**
**T1-W**	No Normalizazion	0.72 (0.67–0.80)	0.73 (0.60–0.80)	0.73 (0.60–0.80)	0.67 (0.61–0.75)	0.73 (0.60–0.77)
	Intensity Normalization	0.80 (0.73–0.83)	0.79 (0.68–0.83)	0.80 (0.68–0.83)	0.80 (0.71–0.83)	**0.82 (0.73–0.86)**
**T2-W**	No Normalization	0.73 (0.66–0.80)	0.72 (0.60–0.825)	0.72 (0.66–0.77)	0.72 (0.60–0.77)	0.67 (0.60–0.77)
	Intensity Normalization	0.77 (0.66–0.86)	0.77 (0.68–0.83)	0.77 (0.66–0.83)	0.73 (0.68–0.80)	0.77 (0.67–0.77)
**T1-W/T2-W**	No Normalization	0.77 (0.67–0.86)	0.73 (0.66–0.83)	0.73 (0.66–0.83)	0.67 (0.60–0.72)	0.73 (0.60–0.80)
	Intensity Normalization	0.80 (0.73–0.86)	0.80 (0.73–0.83)	0.80 (0.72–0.83)	0.77 (0.68–0.83)	0.80 (0.72–0.83)
**OS**	**Clinical Features**	**ETC**	**SVM**	**LR**	**RF**	**KN**
		0.60 (0.44–0.67)	0.71 (0.66–0.79)	0.71 (0.66–0.77)	0.60 (0.54–0.67)	0.67 (0.60–0.77)
**PFS**	**Radiomics Features**	**ETC**	**SVM**	**LR**	**RF**	**KN**
**T1-W**	No Normalizazion	0.67 (0.54–0.72)	0.68 (0.58–0.75)	0.71 (0.66–0.79)	0.60 (0.50–0.72)	0.60 (0.54–0.73)
	Intensity Normalization	0.60 (0.50–0.66)	0.68 (0.60–0.68)	0.68 (0.66–0.73)	0.60 (0.50–0.66)	0.67 (0.55–0.66)
**T2-W**	No Normalization	0.67 (0.55–0.75)	0.67 (0.61–0.68)	0.67 (0.61–0.68)	0.60 (0.50–0.73)	0.67 (0.51–0.73)
	Intensity Normalization	0.68 (0.57–0.76)	**0.80 (0.67–0.88)**	0.80 (0.67–0.86)	0.68 (0.55–0.76)	0.73 (0.67–0.83)
**T1-W/T2-W**	No Normalization	0.67 (0.50–0.74)	0.67 (0.60–0.73)	0.67 (0.58–0.73)	0.60 (0.46–0.67)	0.67 (0.58–0.73)
	Intensity Normalization	0.63 (0.50–0.75)	0.70 (0.60–0.80)	0.73 (0.62–0.80)	0.67 (0.58–0.75)	0.68 (0.60–0.75)
**PFS**	**Radiomics + Clinical Features**	**ETC**	**SVM**	**LR**	**RF**	**KN**
**T1-W**	No Normalizazion	0.60 (0.44–0.67)	0.67 (0.60–0.71)	0.67 (0.55–0.71)	0.60 (0.51–0.73)	0.60 (0.47–0.72)
	Intensity Normalization	0.60 (0.45–0.67)	0.68 (0.61–0.67)	0.68 (0.60–0.67)	0.60 (0.50–0.72)	0.60 (0.45–0.67)
**T2-W**	No Normalization	0.60 (0.48–0.71)	0.61 (0.55–0.67)	0.67 (0.55–0.76)	0.60 (0.50–0.70)	0.62 (0.55–0.67)
	Intensity Normalization	0.68 (0.50–0.76)	0.72 (0.60–0.80)	0.69 (0.60–0.75)	0.70 (0.60–0.77)	0.69 (0.60–0.73)
**T1-W/T2-W**	No Normalization	0.64 (0.55–0.68)	0.67 (0.66–0.71)	0.67 (0.61–0.77)	0.61 (0.50–0.73)	0.61 (0.50–0.67)
	Intensity Normalization	0.61 (0.44–0.68)	0.69 (0.60–0.73)	0.65 (0.55–0.68)	0.60 (0.50–0.67)	0.60 (0.45–0.62)
**PFS**	**Clinical Features**	**ETC**	**SVM**	**LR**	**RF**	**KN**
		0.55 (0.41–0.60)	0.62 (0.51–0.67)	0.67 (0.63–0.71)	0.57(0.47–0.65)	0.55 (0.40–0.61)

## Data Availability

The dataset analyzed in the current study is available from the corresponding author on reasonable request.

## Supplementary Materials

The following supporting information can be downloaded at:https://www.mdpi.com/article/10.3390/bioengineering10030285/s1. Table S1: The SoA of Radiomics Analysis on PCNSL. Table S2: The scanner characteristics for groups of patients examined (All scanners are 1.5 T). For each group, we reported the Ripetition Time (TR), Echo Time (TE), and Flip Angle (FA) for both sequences T1-W and T2-W.
