# Supplementary material for: Radiomics-Based Machine Learning Model for Predicting Overall and Progression-Free Survival in Rare Cancer: A Case Study for Primary CNS Lymphoma Patients
Source: Bioengineering (Basel). 2023 Feb 22;10(3):285. doi: 10.3390/bioengineering10030285 (PMC10045100; doi:10.3390/bioengineering10030285)
Supplement: Supplementary file 1 [file bioengineering-10-00285-s001.zip › bioengineering-2201871-supplementary.pdf]

Table S1.

The SoA of Radiomics Analysis about PCNSL tumor.

Title	DOI	Patients	Data Augumentation	Aim	Tumor Type	Pre-processing Image / Normalize Image method
Diffusion radiomics as a diagnostic model for atypical manifestation of primary central nervous system lymphoma: develompment and multicenter external validation	https://doi.org/10.1093/neuonc/noy021	112 patients (70 glioblastoma/ 42 PCNSL)	NO	To evaluate technical feasibility, diagnostic performance, and generalizability of a diffusion radiomics model for identifying atypical primary central nervous system lymphoma (PCNSL) mimicking glioblastoma.	PCNSL/ GLIOBLASTOMA	Yes WhiteStripe
Comparison of Radiomics-Based Machine-Learning Classifiers in Diagnosis of Glioblastoma From Primary Central Nervous System Lymphoma	https://doi.org/10.3389/fonc.2020.01151	138 patients (76 glioblastoma, 62 PCNSL)	NO	To evaluate the ability of magnetic resonance (MR) radiomics-based machine learning algorithms in differentiating glioblastoma (GBM) from primary central nervous system Limphoma (PCNSL).	PCNSL/ GLIOBLASTOMA	NO
Multiparametric-MRI-Based Radiomics Model for Differentiating Primary Central Nervous System Lymphoma From Glioblastoma: Development and Cross-Vendor Validation	https://doi.org/10.1002/jmri.27344	240 patients (129 glioblastoma/ 111 PCNSL)	NO	To validate the generalization ability of radiomics models based on multiparametric-MRI (MP-MRI) for differentiating PCNSL from GBM.	PCNSL/ GLIOBLASTOMA	Yes Zscore
Radiomic features and multilayer perceptron network classifier: a robust MRI classification strategy for distinguishing glioblastoma from primary central nervous system lymphoma	https://doi.org/10.1038/s41598-019-42276-w	123 patients (73 glioblastoma/ 50 PCNSL)	NO	to establish a high-performing and robust classification strategy, using magnetic resonance imaging (MRI), along with combinations of feature extraction and selection in human and machine learning using radiomics or deep features by employing a small dataset.	PCNSL/ GLIOBLASTOMA	Yes WhiteStripe

Contrast-Enhanced MRI Texture Parameters as Potential Prognostic Factors for Primary Central Nervous System Lymphoma Patients Receiving High-Dose Methotrexate-Based Chemotherapy	https://doi.org/10.1155/2019/5481491	52 patients (PCNSL)	NO	to evaluate the prognostic value of texture features on contrast-enhanced magnetic resonance imaging (MRI) for patients with primary central nervous system lymphoma (PCNSL).	PCNSL	NO
Classification of Primary Cerebral Lymphoma and Glioblastoma Featuring Dynamic Susceptibility Contrast and Apparent Diffusion Coefficient	10.3390/brainsci10110886	74 patients (37 glioblastoma/ 37 PCNSL)	NO	to differentiate primary central nervous system lymphoma (PCNSL) and glioblastoma (GBM) via multimodal MRI featuring radiomic analysis.	PCNSL/ GLIOBLASTOMA	Yes<br>Not cited
Comparison between Glioblastoma and Primary Central Nervous System Lymphoma Using MR Image-based Texture Analysis	10.2463/mrms.mp.2017-0044	60 patients (44 glioblastoma/ 16 PCNSL)	NO	To elucidate differences between glioblastoma (GBM) and primary central nervous system lymphoma (PCNSL) with MR image-based texture features.	PCNSL/ GLIOBLASTOMA	Yes<br>Intensity Standardization
Radiomics features to distinguish glioblastoma from primary central nervous system lymphoma on multi-parametric MRI	https://doi.org/10.1007/s00234-018-2091-4	143 Patients (78 glioblastoma/ 65 PCNSL)	NO	To determine the feasibility of using high dimensional computer-extracted features, known as radiomics features, in differentiating primary central nervous system lymphoma (PCNSL) from glioblastoma on multi-parametric MR imaging including diffusion-weighted imaging.	PCNSL/ GLIOBLASTOMA	NO
A Radiomic Machine Learning Model to Predict Treatment Response to Methotrexate and Survival Outcomes in Primary Central Nervous System Lymphoma (PCNSL)	https://doi.org/10.1182/blood-2020-141941	47 Patients (PCNSL)	NO	To hypothesize that machine learning model using advanced magnetic resonance imaging (MRI) tumor characteristics will improve the accuracy of clinical models to predict response to MTX and survival outcomes.	PCNSL	NO

Table S2. The scanner characteristics for groups of patients examined (All scanners are 1.5 T). For each groups of scanner wereported the Ripetition Time (TR), Echo Time (TE), and Flip Angle (FA) for both sequences T1-W and T2-W.

	**Manufacturer**	**Model**	**TR - T1-W**	**TR -T2-W**	**TE - T1-W**	**TE -T2-W**	**FA - T1-W**	**FA - T2-W**
**Group A**								
23	Philips medical system	Achieva	450-650	4400-6230	12-15	100-120	69-75	90
14	Philips medical system	AchievadStream	579-580	4445-4859	12	100	69	90
14	Philips medical system	Intera	595-600	3000-4438	10-15	80-100	69	90
3	GE medical system	Signa HDx	510-520	5665	12	116	80	90
1	Philips medical system	Ingenia	450	3527	15	100	69	90
1	SIEMENS	MAGNETOM AERA	358	5060	8.9	107	90	150
**Group A2**								
19	Philips medical system	Achieva	450-650	4400-6230	12-15	100-120	69-75	90
11	Philips medical system	AchievadStream	580	4445-4859	12	100	69	90
12	Philips medical system	Intera	595-600	3000-4400	10-15	80-100	69	90
3	GE medical system	Signa HDx	520	5665	12	116	80	90
1	Philips medical system	Ingenia	450	3527	15	100	69	90
1	SIEMENS	MAGNETOM AERA	358	5060	8.9	107	90	150
**Group A1 (First acquisition)**								
7	Philips medical system	Achieva	349-623	3633-6446	12-15	100-120	69-72	90
4	Philips medical system	Ingenia	450-2060	3527-6211	15-20	100-110	69-90	90
3	Philips medical system	AchievadStream	579-580	4454-4859	12	100	69	90
2	Philips medical system	Intera	595-2000	3000-6446	10-15	85-100	69-90	90
2	GE Medical System	Dyscovery MR 450	300-480	7970-8635	20	97-98	90	160
1	GE medical system	Signa HDx	520	5665	12	116	80	90
3	SIEMENS	MAGNETOM AERA	11-584	4000-5000	7.16-8.9	92-99	15-90	125-150
1	SIEMENS	MAGNETOM ESSENZA	2000	4000	9.1	99	150	150
**Group A1 (Second acquisition)**								
13	Philips medical system	Achieva	450-650	4409-6225	12-15	100	69-75	90
7	Philips medical system	AchievadStream	579	4446-4859	12	100	69	90
1	Philips medical system	Intera	599	4438	15	100	69	90
1	SIEMENS	Avanto fit	373	5500	8.4	110	90	150
1	SIEMENS	MAGNETOM AERA	358	5060	8.9	107	90	150
**Group A1 (Third acquisition)**								
10	Philips medical system	Achieva	6.8-596	3003-4454	3.34-15	100	8-69	90
12	Philips medical system	AchievadStream	579-588	4446-4859	12	100	69	90
1	Philips medical system	Intera	599	4438	15	100	69	90
